# Baird-Pattinson Aetiological Classification and Phases of Delay Contributing to Stillbirths in a Nigerian Tertiary Hospital

**DOI:** 10.1155/2016/1703809

**Published:** 2016-01-14

**Authors:** Jacob Olumuyiwa Awoleke, Abiodun Idowu Adanikin

**Affiliations:** Department of Obstetrics and Gynaecology, Ekiti State University, Ado-Ekiti, Nigeria

## Abstract

*Purpose*. This study aims to identify triggers of stillbirth in the study setting and to make suggestions to reduce the prevalence.* Method*. A three-year retrospective case-control study of stillbirths at Ekiti State University Teaching Hospital.* Results*. The stillbirth rate was 33 per 1000 births. Based on Baird-Pattinson classification of the primary obstetric causes of stillbirth, adverse intrapartum events, hypertensive diseases, and unexplained intrapartum fetal deaths were topmost causes of stillbirths. In comparison with the controls, other identified predictors of SB were grand multiparity (*p* = 0.016), delays in seeking medical care and/or in receiving treatment (*p* = 0.001), wrong initial diagnosis (*p* = 0.001), inadequate intrapartum monitoring (*p* = 0.001), and inappropriate clinical management (*p* = 0.001).* Conclusion*. Stillbirth rate remains high in our setting. Elimination of obstacles to accessing care, effective management of hypertensive disorders in pregnancy, updated health facilities, improved dedication to duty, and retraining of health workers will reduce the prevalence.

## 1. Introduction

Sub-Saharan Africa has a high burden of stillbirths (SB), most of which are late preterm, term, and intrapartum deaths [1, 2]. No doubt, these have had tremendous social and health implications on affected families and the society [3]. Unfortunately the trend has continued over the years, despite the fact that about two-thirds of these deaths may be preventable [4–6].

Periodic enquiry into causes of SB and successful implementation of intervention strategies have allowed the developed nations to make giant strides in curbing needless fetal deaths but this has not been the case in Africa [2]. Knowledge forms the pedestal on which interventions rely, and if we will reverse the pathetic pregnancy outcome indices, frequent, robust, and in-depth review of contributors to fetal death becomes inevitable.

What is the current situation now? Is there any departure from what it has been before? What factors presently contribute to SB in our setting and how well can we modify them? Where knowledge of the actual burden and causes of SB is deficient, expectations of preventability will be low and this will negatively impact the level of priority accorded to SB reduction [7]. Therefore, it becomes necessary for each country/region desiring to lower its burden of SB to understand the local causes so that appropriate intervention strategies can be developed and implemented.

Thus, the focus of this study is to identify the triggers for SB in a Nigerian population with the aim of making recommendations to reduce them.

## 2. Method

The setting for this study was the maternity unit of the Ekiti State University Teaching Hospital (EKSUTH), located in the semiurban capital city of Ekiti State, south-western Nigeria. EKSUTH was created on April 1, 2008, from the erstwhile State Specialist Hospital, Ado-Ekiti, and it is the apex health facility in the state serving a population of about 2,398,957 [8]. The hospital also receives referrals from neighbouring Kogi, Osun, and Ondo states. Its average annual delivery rate is 2000/year.

A 3-year review of obstetric records was done from January 2012 to December 2014 and file numbers of patients with SB were identified. The files were subsequently retrieved from the hospital's health information management department. SB was defined as the birth of a baby with a birth-weight of 1000 g or more, 28 weeks' gestation or more, or a body length of 35 cm or more, who died before or during labour [9]. Those with SB therefore formed the study group while inclusion into the control group requires having a live birth. It was decided that, for every case of SB, the next two live births should be chosen as controls. These records were equally retrieved and reviewed for data extraction.

Using a proforma, data were obtained from the SB case files about the maternal age, parity, probable cause of SB based on Baird-Pattinson's classification system [10], documented events potentially contributing to SB before and/or after presenting at the tertiary health facility, and so forth. Corresponding relevant data were also obtained for the controls.

For the purpose of this study, we defined delay in seeking help as not asking for medical assistance >24 hours after first symptom of pregnancy/labour complications. Delay in transfer from referring hospital was defined as not leaving the referring hospital/mission house for the tertiary hospital >1 hour after referral while delay in receiving treatment was defined as not receiving any medical attention at the tertiary hospital >30 mins after presentation.

The extracted data were analysed using the Statistical Software for the Social Sciences (SPSS) package version 17. Frequency tables were generated and statistical comparison of the cases and controls was done using Student's *t*-test or *χ* ^2^ test as appropriate. The level of significance was set at *p* < 0.05.

Approval for the study was given by the Ethics and Research Committee of Ekiti State University Teaching Hospital.

## 3. Results

In the 3-year period, there were 5408 deliveries and 178 SB, giving a crude SB rate of 33 per 1,000 births. Out of the 178 SB cases, 138 (77.5%) were “unplanned transfers” from lower levels of healthcare and mission houses after onset of pregnancy and/or labour complications. However, from the 4467 patients that obtained antenatal care at the study centre, 40 had SB giving a corrected SB rate of 9 per 1,000.

Table 1 shows the age and parity of women in both the study and control groups. Although the age distribution of the women shows no difference in pregnancy outcome, the same cannot be said for their parity. Whereas nulliparous women were likely to have live births (46.3% versus 36.5%; *p* = 0.031), grand-multiparous women have increased likelihood of SB (5.1% versus 1.1%; *p* = 0.016).

Based on Baird-Pattinson classification of causes of fetal deaths (Table 2), intrapartum-related factors topped the list, 46 (25.9%), then hypertensive diseases, 31 (17.4%). A sizeable proportion, 28 (15.7%), also had unexplained intrapartum fetal death while congenital abnormalities were responsible for 12 (6.7%) SB.

As shown in Table 3, 68 (38.2%) women in the SB group sought medical assistance only after 24 hours of onset of pregnancy/labour complications, 9 (5.1%) stayed for more than 1 hour at the referring hospital/mission house before presenting at the tertiary hospital, and 2 (1.1%) were administered intramuscular oxytocin injection by the referring centre. Delay in seeking medical care however attained statistical significance (38.2% versus 3.9%; *p* = 0.001) in contributing to SB.

Following presentation at the tertiary hospital, 26 (14.6%) women in the SB group did not receive any medical assistance for more than 30 minutes after arrival. Although further enquiry showed that 18 out of the 26 already had fetal demise on arrival, 8 patients with delayed intervention had a live baby at presentation.

Ten (5.6%) patients had an initial wrong diagnosis, clinical management was inappropriate for 18 (10.1%) patients, and in another 20 (11.2%) intrapartum monitoring was poor. When compared with the control group, these factors, delay in receiving treatment (14.6% versus 0%; *p* = 0.001), wrong diagnosis (5.6% versus 0.6%; *p* = 0.001), inadequate intrapartum monitoring (11.2% versus 0.3%; *p* = 0.001), and inappropriate clinical management (10.1% versus 0.8%; *p* = 0.001), significantly contributed to SB in the study group.

## 4. Discussion

The SB rate of 33 per 1000 births as observed in this study is comparable to an average rate of 32 per 1000 births quoted for Low-Middle Income Countries (LMIC) [11]. The figure is however significantly lower than earlier values of 170 per 1000 in Birnin Kudu, northwest Nigeria and 74 per 1000 births in Enugu, Southeastern Nigeria [11, 12]. This is not too surprising as Southwest Nigeria is the most educated region in the country and is known to have better health indices. But the SB rate is still unacceptable when compared to 2–5 per 1000 births in some developed countries [1].

Although various researches have shown increased risks of SB at extremes of parity [13–15], this study only found parity ≥5 as a significant predictor. It is documented that grand-multiparous women rarely access prenatal care because they feel overly confident of their past experience of pregnancy and therefore assume that no untoward event will occur [15]. As much as discouraging high parity is needful, it is important through continuous health advocacy to reinforce the gains of antenatal care irrespective of a woman's parity and/or previous pregnancy experience.

Our analysis indicated that intrapartum-related factors were the commonest cause that led to fetal deaths in the population studied. Ezugwu et al. [11] and Lawn et al. [16] earlier noted that 60% of SB in West Africa are intrapartum-related. It is a reflection of sustained suboptimal levels of intrapartum care in the subregion. However, the achieved low level of intrapartum stillbirths in high-income countries suggest that intrapartum stillbirths are largely preventable with a quality care which includes prompt recognition and management of intrapartum complications [17]. With about one million stillbirths occurring annually during labour, mostly from countries with absent, inadequate, or delayed obstetric care [17], prioritizing access to safe, comprehensive and basic emergency obstetric services will make a great impact on SB rates in such settings.

As found in the index study, the World Health Organization (WHO) identified hypertensive disorder as a leading cause of fetal deaths in developing countries [18]. Though stratified analysis showed that calcium supplementation led to an important reduction in maternofetal deaths attributable to hypertensive disease in pregnancy in that study [18], routine calcium supplementation is not practiced in our setting. Exploring this option may be a worthwhile venture.

More than a tenth of SB was unexplained because specific cause could not be found. Although this appears to be due to absent or inadequate investigations, unknown causes of SB might lead to superstition and speculation. Globally, one in three SB is attributed to the mother's sins and faults, bad luck, or the work of evil spirits [19]. Better societal insight into the medical causes of SB has been linked with improved support for the bereaved [20, 21], thereby underscoring the need for facilities for detailed investigations and autopsy evaluation of all SB in developing countries.

The contribution of delay in seeking care to SB reached significant level in this study. More women with SB failed to ask for medical help early. This possibly found root in poverty, low level of literacy, and failure to recognize warning signs of imminent danger [22–25]. There is a need to sustain efforts/campaigns at educating women, both in and outside the hospital, on how to recognize signs of pregnancy complications. Workable, locality-specific, birth-preparedness scheme is also inevitable if lowering SB rate will be achieved in our environment.

Though sad, the fact remains that despite scaling many hurdles to access quality healthcare, some of the women still experienced delay in receiving medical intervention on arrival at the tertiary centre. Factors contributing to delays in the timely provision of needed care have been frequently mentioned in the literature [22]. These include problems with electricity, lack of sterile materials, lack of essential drugs, challenges in getting cross-matched blood, delay in arrival of anaesthetist or senior obstetrician, patient's refusal to consent to planned action of management, and so forth [22, 26]. These issues prevailed in our study too. But, they are largely preventable. If an integrated and coordinated approach to health care delivery is ensured, it will reduce these problems to the barest minimum.

Shortage of trained personnel and dilapidated health facilities coupled with lack of commitment on the part of health workers make poor clinical management and defective intrapartum monitoring significant triggers for SB. Other studies have reported associations between cases of clinical mismanagement and adverse pregnancy outcomes [26–28]. However, an observational evidence has suggested that widespread use of cardiotocography with prompt recourse to caesarean section for cases of fetal distress will significantly lower SB rates [29]. In developing countries, we need to take a cue from the evidence by ensuring availability of facilities for electronic fetal monitoring and an enabling environment to undertake caesarean section within the shortest possible time. Positive attitudinal change by health workers to their expected duty is sacrosanct while regular emergency drills must be ensured for maternity care providers.

The use of a standard classification system for the causes of SB and a detailed review of the case records to identify prevailing events that contributed to the fetal deaths lend credence to this research. Although the extent of information retrieval is limited in a retrospective study, its adoption in our review is appropriate.

In conclusion, the SB rate in our environment remains high. Grand multiparity, hypertensive disease, intrapartum-related events, and delays in seeking and/or accessing care were major contributing factors. It is essential to reinforce the gains of antenatal care irrespective of a woman's parity and/or previous pregnancy experience. Implementing routine calcium supplementation in our setting may assist in reducing fetal mortality associated with hypertensive disorders in pregnancy. Furthermore, updated health facilities, locality-specific birth preparedness scheme, improved dedication of health workers to duty, and regular retraining of maternity care providers will go a long way to reduce SB in our society.

## Figures and Tables

**Table 1:** Maternal age and parity^a^.

Characteristics	Stillbirth group (*n* = 178)	Control group (*n* = 356)	*p* value
*Age (years)*			
≤19	2 (1.1)	1 (0.3)	0.259
20–34	143 (80.3)	297 (83.4)	0.377
≥35	33 (18.5)	58 (16.3)	0.515
*Parity*			
0	65 (36.5)	165 (46.3)	0.031^*∗*^
1–4	104 (58.4)	187 (52.5)	0.197
≥5	9 (5.1)	4 (1.1)	0.016^*∗*^

^a^Values are given as *n* (%); ^*∗*^significant at *p* < 0.05.

**Table 2:** Baird-Pattinson classification of causes of fetal death, *n* = 178.

Cause	Frequency (%)
Antepartum hemorrhage	20 (11.2)
Unexplained intrapartum fetal death	28 (15.7)
Spontaneous Preterm labour	5 (2.8)
Intrapartum related	46 (25.9)
Infection	5 (2.8)
Fetal abnormalities	12 (6.7)
Hypertensive disorders	31 (17.4)
Maternal diseases	6 (3.4)
Intrauterine growth restriction	1 (0.6)
Unknown	24 (13.5)

**Table 3:** Events potentially contributing to stillbirth.

Events	Stillbirth group (*n* = 178)	Control group (*n* = 356)	*p* value
Before presenting in tertiary hospital			
Delay in seeking medical help	68 (38.2)	14 (3.9)	0.001^*∗*^
Delay in transfer from referring hospital	9 (5.1)	8 (2.2)	0.081
Injection of bolus oxytocin during labour by referring hospital/mission house	2 (1.1)	4 (1.1)	>0.999
After presenting in tertiary hospital			
Delay in receiving treatment at the tertiary hospital	26 (14.6)	0 (0)	0.001^*∗*^
Wrong diagnosis	10 (5.6)	2 (0.6)	0.001^*∗*^
Inadequate intrapartum monitoring	20 (11.2)	1 (0.3)	0.001^*∗*^
Inappropriate clinical management	18 (10.1)	3 (0.8)	0.001^*∗*^

^*∗*^Significant at *p* < 0.05.
